# Der posteriore tibiale Slope als intrinsischer Risikofaktor: Biomechanische Grundlagen, diagnostische Herausforderungen und sagittale Korrektur – Eine Literaturübersicht

**DOI:** 10.1007/s00132-025-04750-4

**Published:** 2025-12-05

**Authors:** Kai von Schwarzenberg, Luis Göker, Tamara Babasiz, Julius Klaßen, Till Rosenkranz, Peer Eysel, Jörgen Hoffmann

**Affiliations:** Medizinische Fakultät und Universitätsklinikum Köln, Klinik und Poliklinik für Orthopädie, Unfallchirurgie und Plastische Chirurgie, Universität zu Köln, Joseph-Stelzmann-Straße 24, 50931 Köln, Deutschland

**Keywords:** VKB-Verletzungen, Biomechanik, Kniegelenk, Meniskus, Osteotomie, ACL injuries, Biomechanics, Knee joint, Meniscus, Osteotomy

## Abstract

**Hintergrund:**

Der posteriore tibiale Slope (PTS) ist die Neigung des Tibiaplateaus in der Sagittalebene und ein wichtiger Faktor für die Biomechanik des Kniegelenks. Ein normaler PTS liegt zwischen 6 und 12°. Abweichungen, wie ein steilerer Slope (> 12°) oder ein flacherer Slope (< 5°), sind mit charakteristischen biomechanischen Veränderungen verbunden.

**Fragestellung:**

Die Arbeit befasst sich mit der klinischen Bedeutung des sagittalen Alignments und dessen Auswirkungen auf das Verletzungsrisiko von Kreuzbändern, Menisken und Knorpel. Außerdem werden gängige radiologische Messmethoden und chirurgische Korrekturen bei pathologischen PTS-Werten untersucht.

**Material und Methoden:**

Die Arbeit fasst Erkenntnisse aus zahlreichen biomechanischen und klinischen Studien zusammen. Die radiologische Bestimmung des PTS sowie die chirurgischen Korrekturmöglichkeiten werden detailliert vorgestellt.

**Ergebnisse:**

Ein erhöhter PTS ist ein signifikanter Risikofaktor für VKB-Rupturen und Transplantatversagen nach Rekonstruktionen. Ein Wert von > 12° hat sich in vielen Studien als statistischer Schwellenwert herauskristallisiert, ab dem das Risiko relevant steigt. Erhöhte Slope-Werte sind zudem mit einem erhöhten Risiko für Meniskuswurzel- und Rampenläsionen assoziiert. Die Evidenz für einen Zusammenhang mit Knorpelschäden ist noch gering. Umgekehrt erhöht ein flacherer Slope (< 5°) das Risiko für Rupturen und Re-Rupturen des HKB. Die Korrektur des PTS durch eine „Closed-wedge“-Osteotomie wird in der Literatur bei Werten > 12° häufig diskutiert und scheint das Risiko einer erneuten VKB-Ruptur zu reduzieren.

**Diskussion:**

Die starke Korrelation zwischen dem sagittalen Alignment und der Bandinstabilität unterstreicht die zentrale Bedeutung des PTS in der Kniegelenkbiomechanik. Folglich muss der PTS als primärer Risikofaktor systematisch in die Diagnostik einbezogen werden. Bei VKB- oder HKB-Transplantatversagen und gleichzeitig pathologischem Slope sollte die operative Korrektur als kausal ansetzende therapeutische Option diskutiert werden, um die Belastung des Transplantats zu minimieren. Hochwertige prospektive Studien sind jedoch notwendig, um die langfristige Überlegenheit dieses kombinierten Ansatzes abschließend zu belegen.

## Abstract (en)

**Background:**

The posterior tibial slope (PTS) is the inclination of the tibial plateau in the sagittal plane and is an important factor for knee joint biomechanics. A normal PTS lies between 6 and 12°. Deviations, such as a steeper slope (> 12°) or a flatter slope (< 5°), are associated with characteristic biomechanical changes.

**Objective:**

This paper addresses the clinical significance of sagittal alignment and its effects on the risk of injury to cruciate ligaments, menisci, and cartilage. Furthermore, common radiological measurement methods and surgical corrections for pathological PTS values are investigated.

**Material and methods:**

The work summarizes findings from numerous biomechanical and clinical studies. The radiological determination of the PTS, as well as the surgical correction options, are presented in detail.

**Results:**

An increased PTS is a significant risk factor for ACL (anterior cruciate ligament) ruptures and graft failure after reconstruction. A value of > 12° has emerged as a statistical threshold in many studies, above which the risk increases significantly. Increased slope values are also associated with an elevated risk of meniscal root and ramp lesions. The evidence for an association with cartilage damage is still low. Conversely, a flatter slope (< 5°) increases the risk for PCL (posterior cruciate ligament) ruptures and re-ruptures. The correction of the PTS through a closed-wedge osteotomy is frequently discussed in the literature for values > 12° and appears to reduce the risk of a renewed ACL rupture.

**Discussion:**

The strong correlation between sagittal alignment and ligament instability underscores the central importance of the PTS in knee joint biomechanics. Consequently, PTS must be systematically included in the diagnostic workup as a primary risk factor. In cases of ACL or PCL graft failure combined with a pathological slope, operative correction should be discussed as a causal therapeutic option to minimize graft strain. However, high-quality prospective studies are necessary to conclusively prove the long-term superiority of this combined approach.

## Einleitung

Obwohl Weichteilstrukturen unzweifelhaft zur Kinematik des Kniegelenks beitragen, zeigt eine zunehmende Anzahl an Studien die wichtige Rolle der knöchernen Morphologie der proximalen Tibia, wobei insbesondere der posteriore tibiale Slope (PTS) sich als kritischer Faktor herauskristallisiert hat [25, 55].

Der PTS wird definiert als die posteriore Neigung des Tibiaplateaus relativ zur Längsachse der Tibia in der Sagittalebene [11]. Laut verfügbarer Literatur liegt der normale PTS je nach Bildgebungsverfahren und gewählten anatomischen Referenzpunkten zwischen 6 und 12° [42, 44, 61]. Auf lateralen Röntgenaufnahmen gilt ein Slope > 12° typischerweise als pathologisch steil [31]. Umgekehrt werden Werte < 5°, bzw. negative Werte, als flach oder revers bezeichnet und sind mit charakteristischen biomechanischen Veränderungen assoziiert [67].

## Radiologische Bestimmung des Slopes

**Abb. 1:**
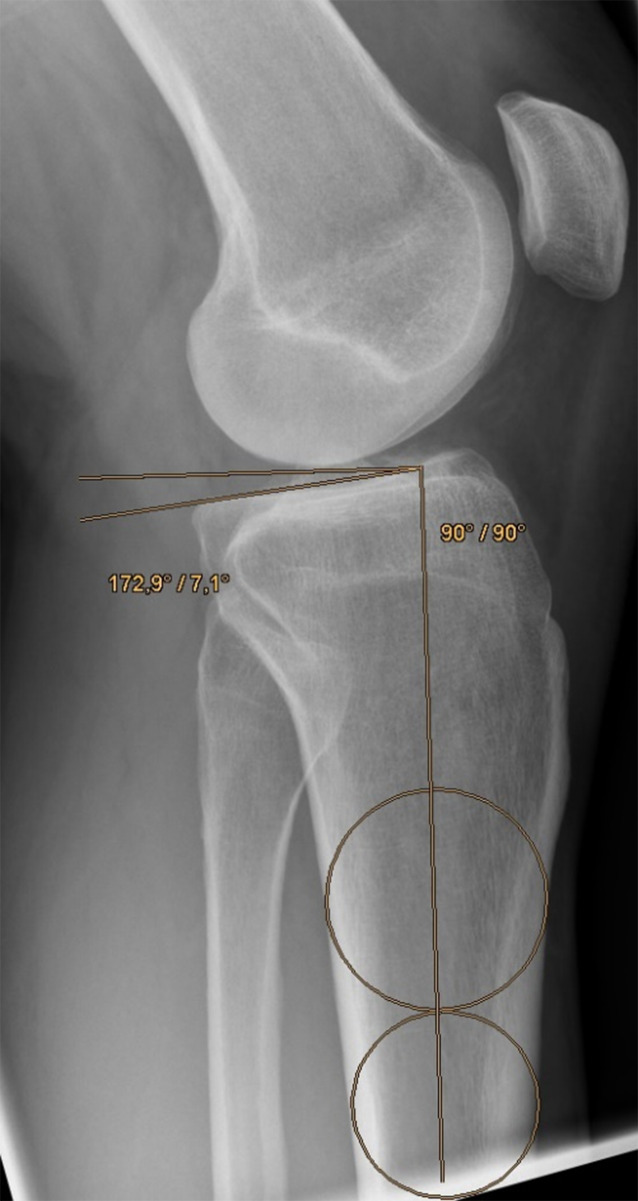
Radiologische Messung des posterioren tibialen Slopes am lateralen Knie-Röntgenbild

Der PTS kann mittels lateraler Röntgenaufnahmen, Magnetresonanztomographie (MRT) oder Computertomographie (CT) gemessen werden [23]. Eine häufig genutzte radiologische Technik, beschrieben von Dejour et al., beinhaltet das Zeichnen zweier konzentrischer Kreise 5 cm und 10 cm distal der Gelenklinie entlang des Tibiaschafts (Abb. 1). Eine Linie, die die Mittelpunkte dieser Kreise verbindet, definiert die proximale anatomische Tibiaachse, und der PTS wird als Winkel zwischen einer Tangente über das Tibiaplateau und einer Senkrechten zur Achse berechnet [11].

Während laterale Röntgenaufnahmen in der klinischen Praxis weit verbreitet sind, bergen sie Fehlerquellen, da die Erfassung der wahren Tibiaschaftachse schwierig ist und die Rotation des Röntgenbildes das Ergebnis beeinflussen kann [23]. Bei Röntgenaufnahmen, bei denen nur kürzere Abschnitte des Tibiaschaftes abgebildet sind, können fehlerhafte Messwerte erhoben werden [18]. Ein Unterschied von bis zu 3° kann aus der Messung des PTS am ganzen Unterschenkel im Vergleich zur Messung am Röntgen mit nur der halben Tibia resultieren [49]. Die höchste Übereinstimmung zwischen der Messung des ganzen Unterschenkels und der nur teilweise abgebildeten Tibia findet sich, wenn der abgebildete Tibiaschaft zwischen 16 und 20 cm lang ist [18]. Eine Malrotation der Femurkondylen führte ebenfalls zu falschen Messwerten [37]. Der Messfehler kann minimiert werden, wenn auf einen posterioren Kondylenabstand von < 5 mm geachtet wird [23]. Zur Bestimmung des wahren tibialen Slopes sollte eine Aufnahme des ganzen Unterschenkels herangezogen werden. Ist diese im klinischen Alltag nicht verfügbar, ist auf das Einhalten der genannten Qualitätskriterien in der seitlichen Röntgenaufnahme des Knies bei der Bestimmung des tibialen Slopes zu achten (Abb. 1).

Im klinischen Alltag stellt die korrekt eingestellte laterale lange Unterschenkelaufnahme das Standardverfahren zur Beurteilung des PTS dar. Allerdings unterliegen konventionelle Röntgenbilder, wie oben beschrieben, systematischen Messfehlern durch Rotationsartefakte der Tibia. Für die präziseste Beurteilung wird die CT als Goldstandard betrachtet, da sie eine detaillierte 3‑D-Analyse ermöglicht, welche Rotationsfehler eliminiert und eine separate Messung des medialen und lateralen tibialen Slopes erlaubt [64]. Darüber hinaus wurden für die MRT verschiedene Techniken zur Bestimmung des tibialen Plateauwinkels (PTA) beschrieben. Hudek et al. beschrieben eine Methode, bei der die Mittelpunkte zweier Kreise in der proximalen Tibia genutzt werden. Der proximale Kreis berührt die Gelenkfläche sowie die anterioren und posterioren Kortizes, der distale Kreis berührt die anterioren und posterioren Kortizes, wobei dessen Mittelpunkt auf dem Radius des proximalen Kreises liegt. Dies erfolgt in der zentralen sagittalen Schicht, in der der tibiale Ansatz des hinteren Kreuzbandes sowie die Eminentia intercondylaris sichtbar sind [28].

Dabei ist es wichtig zu erwähnen, dass es signifikante Unterschiede in den Mittelwerten des tibialen Slope gibt, je nachdem, welche Kombination aus Bildgebungsmodalität (Röntgen, MRT, CT) und spezifischer Messmethode verwendet werden. Die Messwerte können sich zwischen den jeweiligen radiologischen Verfahren um bis zu 5,4° unterscheiden [46]. Dementsprechend ist große Sorgfalt bei der Interpretation klinischer Entscheidungen basierend auf individuellen Messungen geboten. Dies ist auch bei der Bewertung von Studien relevant. In der Regel bezieht sich die Literatur auf Messwerte, welche anhand von Röntgenbildern erhoben wurden. Gängige Referenzwerte für den tibialen Slope werden mit 6° [30], 8,2° [28] oder 9° [11] angegeben.

## Biomechanik

**Abb. 2:**
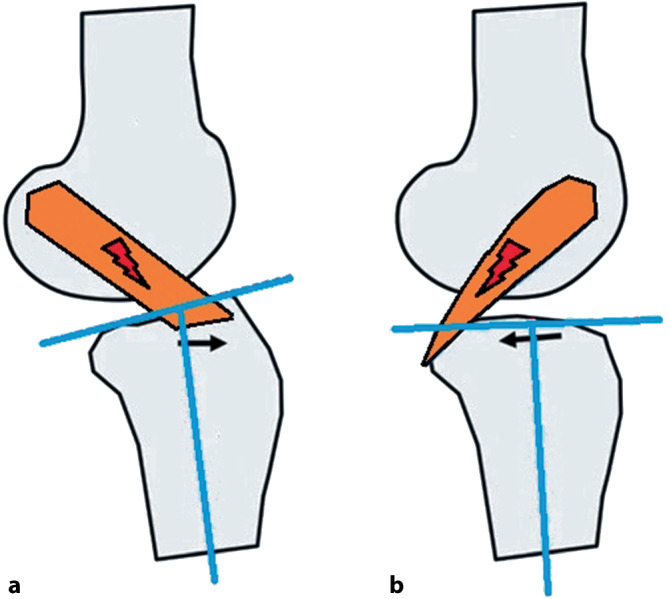
Biomechanische Auswirkung des sagittalen Profils: Ein erhöhter posteriorer tibialer Slope (PTS) führt zu vermehrter anteriorer Translation und Stress auf das vordere Kreuzband (**a**) und ein erniedrigter PTS führt zu vermehrter posteriorer Translation und Stress auf das hintere Kreuzband (**b**)

Veränderungen des tibialen Slopes wirken sich nachweislich auf die Gelenkkinematik und den Knorpeldruck aus [1, 20, 26]. Der PTS spielt eine zentrale Rolle bei der Lastverteilung und Spannungsverteilung in den Kreuzbändern. Mehrere experimentelle Studien konnten durch eine Erhöhung des PTS eine anteriore Translation der Tibia und einen damit verbundenen Anstieg der Spannung des VKB nachweisen [2, 19, 20]. Biomechanisch führt ein steilerer Slope zu einer erhöhten posterior geneigten Stellung des Tibiaplateaus, was unter axialer Belastung die beschriebene verstärkte anteriore Translation der Tibia begünstigt (Abb. 2). Neben der signifikant vermehrten anterioren Translation [1, 20, 26] führt dies ebenso zu einer veränderten Druckverteilung im Gelenk, wobei letzteres sich vor allem auf das laterale Tibiaplateau auszuwirken scheint [26]. Umgekehrt bewirkt ein niedrigerer Slope ein erhöhtes Risiko für Rupturen und Re-Rupturen des hinteren Kreuzbandes bzw. posteriore Instabilitäten nach HKB-Rekonstruktion [47]. Biomechanische Studien zeigen, dass ein geringerer PTS eine posteriore tibiale Translation und somit eine Zunahme der Spannung des HKB bedingt [3, 4].

Der PTS wird oft als Durchschnittswert betrachtet. Neuere biomechanische und klinische Studien betonen jedoch die separate Rolle des medialen posterioren tibialen Slopes (MPTS) und des lateralen posterioren tibialen Slopes (LPTS). Typischerweise ist der LPTS in der Normalbevölkerung etwas flacher als der MPTS [63]. Eine zu große Asymmetrie zwischen den beiden Kompartimenten (Slope-Asymmetrie) oder ein übermäßig steiler LPTS sind für die Kniekinematik besonders relevant [66]. Biomechanisch führt ein steilerer LPTS zu einer vermehrten anterioren Translation des lateralen Tibiakompartiments unter Belastung im Vergleich zum medialen Kompartiment [26]. Diese ungleichmäßige Translation und der dadurch entstehende Scherkraftanstieg im lateralen Kompartiment erhöhen nicht nur die Belastung des Vorderen Kreuzbandes (VKB), sondern gelten auch als signifikanter Risikofaktor für laterale Meniskuswurzelrisse und Knorpelschäden im lateralen Kompartiment [16, 33]. Diese Asymmetrie kann somit zu einer rotatorischen Instabilität beitragen und das Transplantatversagen nach VKB-Rekonstruktion begünstigen [36, 65, 66].

## Erhöhter Slope als Risikofaktor für Rupturen/Re-Rupturen des VKB

Die beschriebenen biomechanischen Erkenntnisse über die vermehrte Spannung auf dem VKB aufgrund der vermehrten Translation konnten auch in klinischen Studien nachgewiesen werden. In den Analysen von Wang et al. (Metaanalyse) und Jagadeesh et al. (randomisiert-kontrolliert) zeigte sich ein Zusammenhang zwischen einem erhöhten PTS und primären Rupturen des VKB [32, 60]. Weiterführend konnte in mehreren Studien ein signifikant erhöhtes Risiko für Re-Rupturen nach primärer VKB-Plastik festgestellt werden, unabhängig von der Transplantatwahl (Quadrizepssehne, Patellasehne oder Hamstrings) [6, 8, 15, 24]. Insbesondere bei jungen Patienten konnte gezeigt werden, dass ein PTS > 12° einen signifikant erhöhten Risikofaktor darstellt. Salmon et al. identifizierten in ihrer 20-Jahres-Studie, dass Patienten unter 20 Jahren mit einem PTS > 12° ein 11-fach höheres Risiko für ein Transplantatversagen nach VKB-Rekonstruktion aufwiesen. Für die Gesamtpopulation war dieser Risikofaktor je nach Altersgruppe mit einer Erhöhung des Rupturrisikos um das 2‑ bis 3‑Fache ebenfalls signifikant, aber deutlich geringer.

## Erniedrigter Slope als Risikofaktor für Ruptur/Re-Rupturen des HKB

Für den Zusammenhang zwischen Rupturen des HKB und dem PTS wird in der Literatur ein antiproportionaler Trend beschrieben. Klinisch manifestiert sich dies durch ein erhöhtes Risiko für primäre Rupturen des HKB und Re-Rupturen nach einer HKB-Plastik bei erniedrigtem PTS [9, 47]. Nedaie et al. nennen in ihrem Review als kritischen Schwellenwert für ein erhöhtes Rupturrisiko einen PTS von ≤ 3,93° [47].

Die klinische Relevanz dieses Zusammenhangs wird dadurch untermauert, dass anteriore „Open-wedge“-Osteotomien zur Vergrößerung des PTS bei HKB-Plastiken gute postoperative Resultate liefern [10].

## Erhöhter Slope als Risikofaktor für Meniskusläsionen

Mehrere Studien deuten darauf hin, dass ein erhöhter PTS nicht nur mit einer erhöhten Inzidenz von Kreuzbandrupturen assoziiert ist, sondern auch als Risikofaktor für Pathologien der Menisken betrachtet werden kann.

Mehrere Studien, darunter auch Systematic Reviews, über den medialen Meniskus belegen, dass ein erhöhter PTS, insbesondere in Form eines erhöhten medialen PTS (MPTS), die auf die mediale Meniskuswurzel einwirkenden Scherkräfte erhöht und somit zu einem Anstieg der Inzidenz von Wurzelrissen des medialen Meniskus führt [16, 40, 45, 50].

Analog hierzu lässt sich für den lateralen Meniskus ein Zusammenhang zwischen Wurzelrissen und einem erhöhten lateralen PTS (LPTS) erkennen. Dieser Zusammenhang wird in der Mehrzahl der Studien jedoch primär in Kombination mit Rupturen des VKB beschrieben [5, 34, 39, 41]. Dzidzishvili et al. identifizierten in ihrem systematischen Review mit insgesamt 1313 Patienten jedoch einen erhöhten PTS auch unabhängig von einer vorderen Kreuzbandruptur als eigenständigen Risikofaktor für meniskale Wurzelrisse [16].

Neben dem vermehrten Auftreten von Wurzelausrissen wird in der Literatur eine erhöhte Inzidenz von Rampenläsionen des medialen Meniskus beschrieben. Diese lassen sich ebenfalls auf die verstärkte anteriore Translation der Tibia zurückführen und können bei einem erhöhten PTS sowohl isoliert als auch in Kombination mit einer Ruptur des VKB auftreten [5, 33].

Darüber hinaus wurde in einer weiteren Studie ein erhöhter tibialer Slope als Risikofaktor für isolierte Meniskusläsionen, unabhängig von der Art der Meniskusverletzung, auch bei ligamentär stabilen Knien identifiziert [54].

## Erhöhter Slope als Risikofaktor für Knorpelläsionen

Eine axiale Fehlstellung in der Frontalebene ist ein wesentlicher Risikofaktor für Knorpelschäden im Knie [29]. Es gibt allerdings auch erste Hinweise, dass die veränderte sagittale Ebene, also ein erhöhter tibialer Slope, zu Knorpelschäden im Knie beitragen kann [27, 38]. Die bereits beschriebenen biomechanischen Veränderungen scheinen vor allem den Knorpel das lateralen Gelenkkompartiments [38] und des patellofemoralen Gelenkes [27] auf unphysiologische Weise zu beanspruchen und zu entsprechender Abnutzung des Knorpels zu führen. Im Gegensatz zur gut belegten Rolle des erhöhten tibialen Slopes als Risikofaktor für Verletzungen der Kreuzbänder und Menisken ist die Evidenzlage hinsichtlich eines Zusammenhangs mit Knorpelschäden bislang jedoch noch gering.

## Öffnende/schließende Osteotomien der proximalen Tibia zur Behandlung von Slope-Pathologien

Bei pathologischen Veränderungen des tibialen Slopes kann dieser bei entsprechender Indikation mittels Osteotomie beeinflusst werden.

## „Anterior tibial Closing-wedge“-Osteotomie

Ein erhöhter tibialer Slope gilt im sagittalen Profil als signifikanter Risikofaktor für das Versagen einer vorderen Kreuzbandplastik. Bei einer notwendigen Reoperation nach Transplantatversagen und Berücksichtigung anderer Versagensursachen (z. B. nichtanatomische Bohrkanäle, Trauma) kann daher bei einem tibialen Slope ≥ 12° die Korrektur mittels anteriorer „Closed-wedge“-Osteotomie als Therapieoption in Betracht gezogen werden, um das Risiko einer erneuten Ruptur zu minimieren [13, 14, 51, 57].

Die biomechanischen Überlegungen, wonach ein erhöhter PTS die anteriore Tibiatranslation steigert und die Transplantatspannung des VKB erhöht, bilden die Rationale für die chirurgische Slope-Reduktion. Die Technik der „anterior tibial Closing-wedge“-Osteotomie (ATCWO) wird eingesetzt, um einen pathologisch steilen PTS > 12° zu korrigieren.

Das Ziel der ATCWO ist die biomechanische Wiederherstellung der Kniestabilität, indem die anterioren Scherkräfte im Gelenk reduziert werden, die ursächlich für die VKB-Überlastung sind. Klinische Studien zeigen, dass die Kombination aus VKB-Rekonstruktion und ATCWO bei Patienten mit steilem PTS im Vergleich zur alleinigen VKB-Rekonstruktion zu signifikant besseren klinischen und radiologischen Ergebnissen führt. Dazu zählen eine verbesserte statische Kniestabilität (geringere anteriore Tibiasubluxation) und eine niedrigere Rate an Transplantatversagen [57, 59]. Diese operativen Korrekturen gelten daher als wichtiger Baustein im Umgang mit rezidivierenden oder primären VKB-Rupturen bei anatomisch prädisponierten Patienten.

Kontraindikationen für die „Closed-wedge“-Osteotomie umfassen ein Genu recurvatum mit Hyperextension > 10°, eine HKB-Insuffizienz, ausgeprägtes Genu varus oder eine endgradige Gonarthrose. Ebenso sollte eine Überkorrektur des Slopes auf < 5° vermieden werden, da hierdurch das Risiko für HKB-Verletzungen steigt [13].

Vadhera et al. differenzieren drei mögliche Lokalisationen der anterioren „Closed-wedge“-Osteotomie: oberhalb, auf Höhe oder unterhalb der Tuberositas tibiae (TT) [57].

Die supratuberositäre Technik wird oberhalb der Tuberositas tibiae durchgeführt und gilt oft als der Standardansatz zur isolierten PTS-Korrektur. Der Vorteil liegt in der direkten und präzisen Korrektur nahe der Gelenklinie. Allerdings birgt sie das Risiko, eine bereits bestehende Patella alta zu verstärken. [12, 22]. Zudem kann diese Technik aufgrund des resultierenden kürzeren proximalen Knochenblocks die Plattenfixation kompromittieren und die Länge des tibialen VKB-Tunnels einschränken [43].

Die Osteotomie auf Höhe der TT erfordert die vorherige Durchführung einer Tuberositas-tibiae-Osteotomie (TTO) zur Ablösung des Patellasehnenansatzes. Der entscheidende Vorteil dieser komplexeren Technik ist die Möglichkeit zur gleichzeitigen Korrektur von Torsionsfehlstellungen oder einer lateralisierten Patella. Der Nachteil liegt jedoch in der erhöhten Morbidität aufgrund der TTO. Sie führt fast immer zu einer Patella baja, was die patellofemorale Kinematik negativ beeinflusst und die Rehabilitationszeit verlängert [43].

**Abb. 3:**
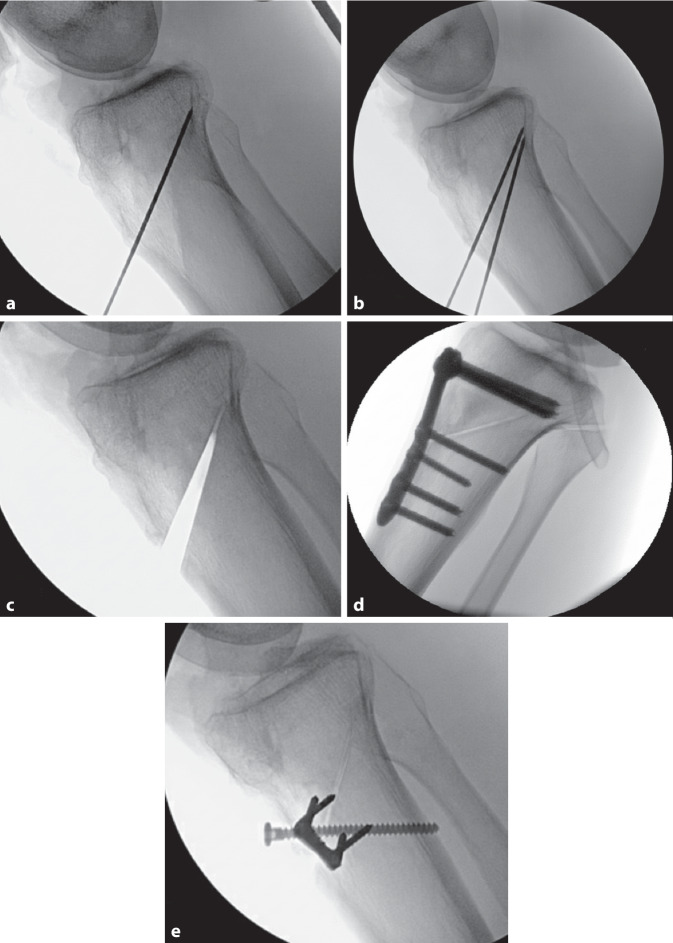
Intraoperative Röntgenaufnahmen mit Positionierung des proximalen K‑Drahtes (**a**) und des distalen K‑Drahtes (**b**). Durchgeführte Osteotomie (**c**) und geschlossener Osteotomiespalt mit Platten- (**d**) oder Schrauben- und Richards-Klemmen-Osteosynthese (**e**)

Die infratuberositäre Technik wird unterhalb der TT durchgeführt, wodurch die Patellasehne nicht beeinträchtigt wird (Abb. 3). Der Hauptvorteil dieser Technik liegt in der Schonung der Patellahöhe, da die Patellasehneninsertion am Hauptsegment der Tibia verbleibt. Somit wird das Risiko einer postoperativen Patella baja oder alta minimiert [43].

Das primäre Ziel der Korrektur ist die Rückführung des tibialen Slopes in den anatomisch und biomechanisch optimalen Normbereich. Um die Transplantatspannung des VKB zu minimieren und die Kniestabilität zu maximieren, wird in der chirurgischen Praxis ein Ziel-PTS (Target-PTS) von idealerweise 5–8° angestrebt [43]. Klinische Studien belegen, dass eine Reduktion des PTS um 6–8° ausreichend ist, um einen signifikanten klinischen Nutzen zu erzielen [56, 58]. Dieses Korrekturausmaß führte in entsprechenden Studien zu einer deutlichen Reduktion der anterioren Tibiatranslation (ATT) und der Transplantatversagensrate. Eine Überkorrektur des PTS (Zielwert < 5°) sollte vermieden werden [58], da dies die auf das HKB wirkenden Scherkräfte erhöht und eine hintere Instabilität verursachen kann [48]. Zudem kann ein zu großes Korrekturausmaß die Patellahöhe verändern und zu sekundären Beschwerden im Patellofemoralgelenk führen [17].

Zusammenfassend erfordert die optimale Wahl der Osteotomietechnik und das Korrekturausmaß eine sorgfältige präoperative 3‑D-Planung, um eine Über- oder Unterkorrektur sowie die schwerwiegende Komplikation der Patellahöhenveränderung zu vermeiden.

Ein systematisches Review (16 Studien, 148 Patienten) zeigt, dass die Slope-reduzierende Osteotomie als Ergänzung zur VKB-Rekonstruktion die Versagensrate senkt und die Patientenzufriedenheit erhöht, besonders bei einem tibialen Slope > 12° [56]. Neuere Daten untersuchen die simultane Slope-reduzierende tibiale Osteotomie bei der primären VKB-Rekonstruktion. Eine Fallserie zeigte, dass bei einem PTS ≥ 15° die kombinierte Vorgehensweise die tibiale Subluxation und VKB-Graft-Laxität signifikant reduzierte [59]. Der wissenschaftliche Nachweis für die Überlegenheit einer kombinierten sagittalen Tibia-Osteotomie und Kreuzbandrekonstruktion gegenüber einer alleinigen Bandrekonstruktion ist derzeit weiterhin limitiert. Die vorliegenden positiven Ergebnisse, wie jene von Wang et al., basieren zumeist auf retrospektiven Kohortenstudien (Evidenzlevel III und IV). Trotz methodischer Ansätze wie dem Propensity Score Matching (PSM) können diese Beobachtungsstudien inhärente Risiken wie den Selektions- und Indikationsbias nicht vollständig eliminieren, was die Vergleichbarkeit der Interventions- und Kontrollgruppen erschwert. Eine kausale Schlussfolgerung, dass die zusätzliche Osteotomie die Versagensrate signifikant senkt, ist somit verfrüht. Des Weiteren weisen die meisten Publikationen kaum Langzeitdaten (> 5 Jahre) auf, was eine fundierte Beurteilung der Überlebensrate des Transplantats und der Spätfolgen für die Gelenkmechanik unmöglich macht. Die aktuellen Daten indizieren lediglich eine Assoziation zwischen der Normalisierung des pathologischen PTS und einem tendenziell geringeren Risiko für ein frühes Transplantatversagen; diese Beobachtungen müssen jedoch durch zukünftige randomisiert-kontrollierte Studien mit hohem Evidenzgrad validiert werden.

## Proximale anteriore „Open-wedge“-Osteotomie

Das Ziel der anterioren „Open-wedge“-Osteotomie ist die Erhöhung des PTS. Die Indikation für diesen Eingriff besteht bei symptomatischem Genu recurvatum mit Hyperextension > 15°, posteriorer Instabilität infolge HKB-Insuffizienz oder wiederholtem Transplantatversagen [52, 53]. Kontraindikationen bestehen bei fortgeschrittener Gonarthrose (Kellgren-Lawrence-Grad IV) sowie bei Achsfehlstellungen > 10° Varus/Valgus [53].

Durch die Modifikation des tibialen Plateauwinkels in der Sagittalebene werden die posterioren Stabilisierungsmechanismen des Kniegelenks verbessert. Die Stabilisierung erfolgt durch eine winkelstabile Plattenosteosynthese, optional unterstützt durch allogene Knochenkeile oder Knochenersatzmaterial, um die Konstruktstabilität zu erhöhen und Komplikationen wie Pseudarthrosen oder Korrekturverlust zu reduzieren [26, 53].

Äquivalent zur ATCWO gibt es auch bei der anterioren „Open-wedge“-Osteotomie verschiedene Techniken, die sich wie bei der ATCWO durch die Relation der Osteotomie zur Tuberositas tibiae unterscheiden.

Dabei liegt auch bei PTS-erhöhenden Osteotomien der Vorteil der supratuberösen Osteotomie darin, dass die Patellahöhe durch diese Technik nicht verändert wird [7].

Bei der transtuberositären Osteotomie wird die anteriore „Open-wedge“-Osteotomie mit einer Tuberositasosteotomie (TTO) kombiniert. Eine solche Kombination wird gewählt, wenn zusätzlich zur sagittalen Korrektur (Slope-Erhöhung) die Patellahöhe (bei einer Patella alta) oder die Ausrichtung des Streckapparates durch eine Medialisierung oder Lateralisierung korrigiert werden muss [35].

Eine infratuberositäre Osteotomie wird in der Regel eher bei der ATCWO mit Slope-Reduktion angewandt, da bei dieser Technik die Patellahöhe nicht beeinflusst wird. Im Gegensatz dazu wird bei der infratuberositären Osteotomie mit anteriorer Öffnung der proximale Knochenblock in Relation zur Gelenklinie nach hinten und unten verlagert. Dies führt dazu, dass der Ansatzpunkt der Patellasehne relativ zum Kniegelenk nach unten verschoben wird, was in einer Patella baja resultiert. Daraus entsteht ein Zielkonflikt der Operation. Während die Indikation der Operation bei posteriorer Instabilität oder Genu recurvatum gegeben sein kann, würde die Patella baja die Kniefunktionalität verschlechtern und somit das Operationsergebnis beeinträchtigen. Daher findet bei der anterioren „Open-wedge“-Osteotomie die infratuberositäre Technik keine Anwendung. Aufgrund dessen findet sich auch keine Literatur, die die infratuberositäre Technik zur Slope-Erhöhung behandelt. Die beschriebenen Implikationen lassen sich jedoch aus der bekannten Literatur in Bezug auf die „medial opening wedge“-HTO (MOWHTO) ableiten [21].

Studien zur HKB-Instabilität erwähnen selten konkrete Zielwerte für die Korrektur, wobei in biomechanischen Studien gezeigt wurde, dass eine Erhöhung des PTS um 5° erforderlich ist, um die posteriore Tibiatranslation effektiv zu limitieren [19]. In der klinischen Studie von Gwinner und Dickschas wird ein durchschnittlicher postoperativer Slope von 11,5° bei einem durchschnittlichen präoperativen Slope von 3,7° angegeben [62].

Generell ist anzumerken, dass aufgrund der deutlich selteneren Durchführung einer anterioren „Open-wedge“-Osteotomie die Literatur noch begrenzt ist, sodass weitere Studien erforderlich sind, um die klinischen Auswirkungen der verschiedenen Osteotomietechniken herauszuarbeiten und das Korrekturausmaß sowie die -zielwerte zu validieren [43].

## Fazit für die Praxis

- Tibialen Slope beachten: Bei Knieinstabilitäten, rezidivierenden Meniskusläsionen oder unklaren Knieschmerzen den tibialen Slope radiologisch bestimmen.
- Erhöhter Slope (> 12°): Erhöhtes Risiko einer Ruptur des vorderen Kreuzbands oder eines Transplantatversagens. Bei jungen, aktiven Patienten frühzeitig kniechirurgische Beurteilung erwägen.
- Abgeflachter Slope (< 5°): An hintere Instabilität oder Insuffizienz des hinteren Kreuzbands denken.
- Richtwerte, keine Cut-offs: 12° und 5° sind Orientierungshilfen; die Indikation ist stets individuell zu stellen.
- Gezieltere Therapie: Kenntnis des Slopes ermöglicht präzisere Indikationsstellung für operative Korrekturen.

## Abkürzungen

ATCWO: Anterior Tibial Closing-Wedge Osteotomy
ATT: Anterior Tibial Translation (Anteriore Tibiatranslation)
CT: Computertomographie
HKB/PCL: Hinteres Kreuzband/Posterior Cruciate Ligament
HTO: High Tibial Osteotomy (Hohe tibiale Osteotomie)
LPTS: Lateral Posterior Tibial Slope (Lateraler posteriorer tibialer Slope)
MPTS: Medial Posterior Tibial Slope (Medialer posteriorer tibialer Slope)
MRI/MRT: Magnetic Resonance Imaging Magnetresonanztomographie
MOWHTO: Medial Opening-Wedge High Tibial Osteotomy
PTS: Posterior Tibial Slope (Posteriorer tibialer Slope)
TT: Tuberositas tibiae
TTO: Tibial Tubercle Osteotomy (Tuberositas-Osteotomie)
VKB/ACL: Vorderes Kreuzband/Anterior Cruciate Ligament
